# Hit it hard: qualitative patient perspectives on the optimisation of immune checkpoint inhibition

**DOI:** 10.1038/s41416-024-02756-x

**Published:** 2024-06-17

**Authors:** Sophie Merrick, Hannah L. Rush, Susanna Daniels, Alison Fielding, Sharon Deveson Kell, Lisa Pickering, Ruth E. Langley, Annabelle South, Duncan C. Gilbert

**Affiliations:** 1 MRC Clinical Trials Unit at UCL, Institute of Clinical Trials & Methodology, 2nd Floor 90 High Holborn, London, WC1V 6LJ UK; 2 Guys and St Thomas NHS Foundation Trust, Great Maze Pond, London, SE1 9RT UK; 3 Melanoma Focus, Salisbury House, Station Road, Cambridge, CB1 2LA UK; 4 Action Kidney Cancer, 11th Floor, 3 Piccadilly Place, Manchester, M1 3BN UK; 5 Royal Marsden Hospital, 203 Fulham Rd, Chelsea, London, SW3 6JJ UK

**Keywords:** Cancer immunotherapy, Oncology

## Abstract

**Background:**

Immune checkpoint inhibitors have transformed the treatment landscape of many cancers, including melanoma and renal cell carcinoma (RCC). Randomised trials are evaluating outcomes from reduced ICI treatment schedules with the aim of improving quality of life, tolerability, and cost-effectiveness. This study aims to provide insight into patient and carer’s perspectives of these trials.

**Methods:**

Seven focus groups were conducted with 31 people with stage IV melanoma, RCC, or caregivers for people receiving ICI. Transcripts were analysed using reflexive thematic analysis.

**Results:**

Three themes were generated: 1) *“Treatment and clinic visits provide reassurance”:* reducing hospital visits may not improve quality of life. 2) *“Assessment of personal risk versus benefit”:* the decision to participate in an ICI optimisation trial is influenced by treatment response, experience of toxicity and perceived logistical benefits based on the individual’s circumstances. *3) “Pre-existing experience and beliefs about how treatment and trials work”*, including the belief that more treatment is better, influence views around ICI optimisation trials.

**Conclusion:**

This study provides insight into recruitment challenges and recommends strategies to enhance recruitment for ongoing ICI optimisation trials. These findings will influence the design of future ICI optimisation trials ensuring they are acceptable to patients.

## Background

Immune checkpoint inhibitors (ICI) have transformed the treatment landscape of many cancers including melanoma and renal cell carcinoma (RCC), resulting in durable responses and long-term survival in patients with advanced cancer [1–3].

ICI were developed using the same principles employed for cytotoxic chemotherapy agents, whereby doses were escalated during early phase studies to establish the maximum tolerated dose, despite a lack of pharmacokinetic, pharmacodynamic, or clinical evidence to justify this approach [4–7].

ICI targeting programmed cell death 1 receptor (PD-1) and its ligand (PD-L1), are routinely administered on a continuous schedule of frequent intravenous administrations (every 2–6 weeks). In advanced disease, they are usually administered for up to two years or until cancer progression or the development of intolerable toxicity [2, 8].

Frequent attendance for treatment and its associated monitoring has the potential to negatively impact patients’ health-related quality of life (HRQoL). Furthermore, ICI can lead to the development of significant life-long immune-related adverse events by activating self-directed immune reactions. Treatment with ICI is placing an increasing financial burden on patients and healthcare services worldwide, with annual costs of these treatments expected to grow from $24 billion in 2022 to $46 billion in 2026 [9]. For individual patients, the financial implications of treatment are associated with poor HRQoL [10–12].

There is increasing evidence that current anti-PD-1/PD-L1 dosing schedules might be optimised [4, 5, 13] and ongoing randomised trials are investigating this via three approaches; early cessation, extended interval or reduced dose, with the aim of improving HRQoL, tolerability, and cost-effectiveness while maintaining efficacy [14–27]. Recruitment to clinical trials investigating de-escalation of cancer treatment can be challenging [28] for example the DANTE trial, which was investigating early cessation of anti-PD-1 ICI in melanoma in the UK, closed prematurely as it did not meet enrolment targets [26, 29]. There is a lack of data regarding patients’ and carers’ perspectives of these approaches and views on participating in ICI optimisation trials. Qualitative research can identify patients’ perspectives to determine the causes of recruitment challenges and develop strategies to overcome these [30–32]. We sought to provide insight into patients’ perspectives of ICI dose scheduling trials, with the aim of developing recommendations to promote recruitment to ongoing projects and to inform the design of future trials.

## Methods

### Design

This qualitative study used focus group discussions (FGDs) to explore patient perspectives of ICI optimisation. Patient and public involvement representatives contributed to the study design including the participant facing materials and FGD topic guide. This study was conducted within a critical realist research paradigm, which takes the viewpoint that research outcomes are influenced by the context in which the research takes place, including the background of the research team [33]. The Standards for Reporting Qualitative Research (SRQR) checklist for this study can be found in the Supplementary Materials, pages 2–4.

### Participants

Participants eligible for this study included people ≥18 years old with stage IV RCC or melanoma or those caring for people receiving ICI. Participants had to be residing in the UK and able to give informed consent.

### Procedure

Advertisements were promoted through cancer charities, support groups and patient forums. Interested individuals were directed to a website with an electronic patient information sheet and access to an eligibility assessment, electronic consent, and background questionnaire. The questionnaire asked for demographic information, cancer diagnosis, distance travelled to receive cancer treatment, and whether they had previously participated in a clinical trial. Participants were purposively selected to ensure that participants with diverse characteristics were included.

During the first FGD, 5 participants’ information given during the introductions was inconsistent with the information provided on the background questionnaire. These individuals had their cameras turned off and did not contribute to the discussion and were therefore excluded from the analysis. Similar issues in qualitative research have been reported in the literature [34–37]. All subsequent participants had a video screening call with the lead investigator (SM) prior to taking part to ensure they were eligible and able to meaningfully contribute to the FGD. The screening approach was successful, and all subsequent participants were fully engaged in the discussion.

FGDs were conducted in person and online via Microsoft Teams at a variety of times, to make them accessible to a range of people, including working professionals, those with caring responsibilities and those less familiar with the technology. The FGDs were guided by a topic guide, which can be found in the Supplementary Materials, pages 5-8.

Separate FGDs were held for participants with RCC, melanoma, and those who were carers. FGDs were held sequentially, allowing for data analysis between discussions. This allowed subsequent discussions to be adapted so that certain topics could be explored in more detail. Sample size decisions were influenced by: (1) the desire to run FGDs in person and online and (2) to ensure a minimum of two FGDs for each of the cancer types and an additional FGD for carers. Data collection continued until no additional concepts were raised.

The FGDs lasted approximately 90 min and were facilitated by two oncologists (SM and HR). At the beginning of each FGD, participants were shown a 10-min presentation on the rationale for ICI optimisation and hypothetical ICI optimisation trial designs (see Supplementary Materials, pages 9–15). There was then an open discussion around perceived barriers and facilitators to participation in each of the hypothetical trials, and factors which influenced their views. FGDs were recorded and transcribed using Microsoft Teams. Transcripts were checked for accuracy before data were anonymised. Participants were reimbursed with a £25 voucher for taking part.

### Analysis

**Fig. 1:**
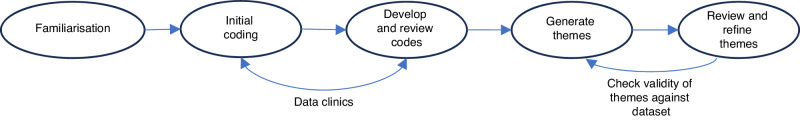
Overview of analysis process.

Data were analysed using reflexive thematic analysis [38]. Figure 1 provides an overview of our analysis process, starting with familiarisation with the data, initial coding, developing and reviewing codes, generating themes and reviewing and refining themes. Reflexive Thematic Analysis acknowledges that the researcher’s knowledge, assumptions, experience, values and pre-existing beliefs will influence their research. Initial coding with a combination of inductive and deductive codes was performed by SM. Excerpts of the transcripts were also reviewed and coded by AS and HR in data clinics. We have sought to ensure that our findings and conclusions are valid through employing an iterative process, where generated themes are compared back to the data and revised as needed, to ensure they are supported by the data. Codes and patterns were refined as more data was analysed. After 7 FGDs had been coded, with 178 codes created, SM clustered codes with a shared meaning (see example of clustered codes in Supplementary Material, page 16). This allowed us to identify patterns between the codes, which we used to derive initial themes and sub-themes. The validity of these initial themes and sub-themes was assessed by re-reviewing the dataset. Codes and themes were discussed and refined at analysis meetings with SM, AS and HR. The thematic structure was then discussed at a meeting with three participants who had taken part in the study, to check the themes made sense to them and to collaboratively develop recommendations.

An ideal type analysis was performed to explore whether certain participant characteristics influenced their views on ICI optimisation [39]. SM developed case reconstructions for each participant and grouped cases into those who were positive, uncertain, or negative about participating in an ICI optimisation trial. To assess credibility, AS independently regrouped the cases and SM and AS discussed and resolved any disagreements. Each group was then compared to determine which factors influenced participants’ views, optimal cases were identified, and ideal-type descriptions developed.

NVivo 12 software was used to manage the data and assist with the analytic process.

## Results

### Sample demographics

64/114 applicants were selected to participate. 21/64 ineligible participants were subsequently excluded following screening. 7/43 who were invited to attend a FGD did not attend. Between January and May 2023 7 FGDs were conducted with 36 participants (3 FGDs for RCC, 3 FGDs for melanoma and 1 for carers). 5 participants from the first FGD (prior to the implementation of the screening video call) were excluded from the analysis as they did not meaningfully contribute to the discussion. The characteristics for the 31 participants included in the analysis are outlined in Table 1. Only 5 participants (2 RCC, 3 melanoma) had previous experience of taking part in a clinical trial.

**Table 1:** Participant characteristics.

		Melanoma	RCC	Carers	Total
		17	11	3	31
Gender	Female	11	3	3	17
	Male	6	8	0	14
Age range	18–25	0	0	0	0
	26–35	1	0	0	1
	36–45	3	1	0	4
	46–55	5	2	1	8
	56–65	5	7	1	13
	66–75	3	1	1	5
	>75	0	0	0	0
Highest level of education	No qualification	0	0	0	0
	GCSE	2	1	1	4
	NVQ	1	0	0	1
	A level	2	1	1	4
	Degree	6	7	0	13
	Higher Degree	4	2	1	7
	Other	2	0	0	2
Ethnicity	White	17	10	3	30
	Mixed	0	0	0	0
	Asian	0	0	0	0
	Black	0	1	0	1
	Other	0	0	0	0
Dependents	Yes	7	2	0	9
	No	10	9	3	22
Time to travel to cancer centre (*n* = 3 carers not included)	<30 min	5	6	NA	11
	≥30 min <1 h	7	3	NA	10
	≥1 h <2 h	4	1	NA	5
	≥2 h	1	1	NA	2
Prior enrolment in a clinical trial (*n* = 3 carers not included)	Yes	2	3	NA	5
	No	15	8	NA	23

### Findings

**Fig. 2:**
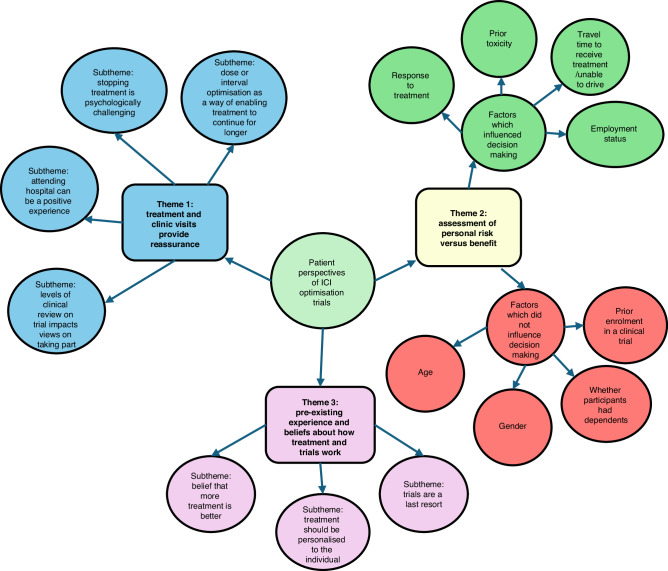
Patient perspectives on ICI optimisation themes and subthemes.

Participants’ perspectives on ICI optimisation were grouped into three themes: (1) “Treatment and clinic visits provide reassurance”, (2) “Assessment of personal risk versus benefit” and (3) “Pre-existing experience and beliefs about how treatment and trials work”. Themes were further divided into subthemes (see Fig. 2). Participants are described by a study ID number, with the preface M for participants with melanoma, R with RCC, and C for carers. Where participants with previous trial experience are quoted, this is indicated following the quote; all other quotes are from participants without previous trial experience.

#### Theme 1: Treatment and clinic visits provide reassurance

One of the main aims of ICI optimisation trials is to improve HRQoL, assuming that a reduction in treatment visits would be desirable for patients. However, in this study, many participants reported that they found attending hospital for treatment or clinic visits reassuring, suggesting that reducing hospital visits may not necessarily improve HRQoL.

##### Subtheme 1: Attending hospital can be a positive experience

Patients receiving ICI reported attending for treatment allowed them to connect with others who were in a similar situation, giving a sense of community and providing support. Some participants described this as particularly beneficial during the COVID-19 pandemic when there was a reduction of in-person support services. Some participants also welcomed the opportunity to unwind and disconnect from external pressures.

“*It’s funny because you would think it’s a depressing time, but actually there is a lot of camaraderie there and they’re really looked after by the teams*.” (C2)

##### Subtheme 2: Stopping treatment is psychologically challenging

Some participants who had stopped treatment reported anxiety about not receiving treatment and feeling abandoned.

“*Well, I found it really hard when I had to stop [treatment] after four [cycles]. From the psychological point of view, I felt, you know, this is just me and you know the gods, really*.” (M4)

Participants expressed concern about a lack of monitoring and contact with their medical team when they stopped treatment, prompting feelings of worry and isolation.

“*When your treatment comes to an end, you feel you’re in a black hole because whilst you’re on treatment, you’re being monitored, you’re being looked after and sort of at the end there’s nothing there*.” (C5)

##### Subtheme 3: Dose or interval optimisation as a way of enabling treatment to continue for longer

When asked about their preferred optimisation approach, most participants preferred dose reduction or extending the interval between treatments over early cessation as they did not want to discontinue treatment and the associated monitoring. Several participants found reducing the dose or extending the interval between treatments attractive, as they felt these dosing schedules would be more tolerable and allow them to continue their overall treatment for longer.

“*If I had been offered a lesser dose as a trial or a bigger gap to recover in the meantime that would have been terrific for me because it was literally all or nothing and I ended up with nothing*. (M6)

##### Subtheme 4: Levels of clinical review on trial impacts views on taking part

Many participants found frequent contact with their specialist team reassuring, particularly early in their diagnosis. Lack of contact with their medical team may be detrimental psychologically.

“*I quite like the idea that I go to hospital once a month. It isn’t a drag for me at all to have blood tests, chat with my oncologists or the nurse. You know, I feel like they’re keeping a watchful eye on me. If I didn’t go in for two months, I think I would start wondering what, you know, if everything is OK*.” (R3)

ICI are typically administered as intravenous infusions every 2–6 weeks. Many participants wanted reassurance that if they received treatment less often, or stopped treatment, they would still receive regular monitoring from their medical team. Most reported having a telephone consultation in between treatment visits would provide adequate reassurance. Participants also felt it was important to have the option to resume standard of care (SOC) treatment if their cancer progressed and that this should be communicated clearly when they were considering whether to enrol.

“*If you’re sort of carefully monitored thereafter. That would be crucial, I think*.” (M7)

“*As somebody said earlier, nobody knows the exact way of doing things and if you say that, OK, we stop [treatment] and it starts growing again, we’ll put you back on it. I’d feel reasonably comfortable about that*.” (R8)

Some participants felt taking part in a clinical trial would increase the monitoring and input from medical staff in comparison to receiving treatment outside of a clinical trial and this would encourage them to take part.

“*I’ve been on the … trial, so that was my only experience of any oncology treatment. So my first experience was I was given a research nurse and I was given access to this research nurse and my bloods were done regularly. I could have a sort of personal hotline to her, but at the time the care and attention that you get when you’re on a trial as opposed to [SOC] and I’m not saying that the care is inferior on the NHS, but it definitely is different being on a trial and not being on a trial*.” (R9 – previous clinical trial experience)

#### Theme 2: Assessment of personal risk versus benefit

All participants reported they would prefer to receive optimised treatment if these regimens were proven to be equally as effective within a clinical trial. However, participants differed in their views about whether they would participate in an ICI optimisation trial. An ideal type analysis was conducted to determine which factors influenced participants’ views (see supplementary materials for optimal cases). This suggested that people’s willingness to participate was influenced by four main personal factors which participants used to assess the personal risk versus benefit of participating: response to treatment, prior toxicity, travel time to receive treatment/unable to drive and employment status. Of these factors, the most important were response to treatment and prior toxicity. Age, gender, whether participants had dependents and prior participation in a clinical trial did not appear to influence decision making.

##### Response to treatment

In general, participants were more positive about participation when they had experienced a good response to treatment, particularly if it was a complete response. Many participants indicated that they would be reluctant to participate in an early cessation trial unless they had a complete response.

“*Personally, if the scan was clear, I wouldn’t have a problem with it [enrolling in an early cessation trial]. If the scan wasn’t clear, I’d be very reluctant to come off. If it was working, but not to the point of clear, I wouldn’t want to be coming off it after a year. Even with the chance of going back on it*.” (R10)

##### Prior toxicity

Although many of the participants who responded well to treatment were positive about participating in an ICI optimisation study, others were reluctant to change the dose or schedule of a treatment they perceived was working well for them. This was particularly evident in those who were tolerating treatment well with minimal toxicity.

“*I was doing well with minimal side effects… If it’s not broke, don’t fix it*.” (M12)

In contrast, participants who experienced toxicity were more motivated to participate, as the primary driver for participating in an ICI optimisation trial was a potential reduction in toxicity.

“*You know, if it’s still going to sort of do the job, but it’s not going to send the liver mad then I would want a bit of that*.” (R10)

“*My thoughts are more to do with, I think, management of side effects [rather than logistical benefits]. We did have a conversation about having a shorter period on nivolumab. I know that there is a trial run at [cancer centre] I think that runs that basically you can have a nivolumab for a year instead of two and then you’re being monitored and at the time we were willing to do that. We were actually welcoming the opportunity*.” (C38)

##### Travel time to receive treatment and employment status

Some participants felt that attending less often for treatment would have logistical benefits, including spending more time with family, spending less time travelling to hospital and minimising the impact of treatment on work. Participants who had to travel long distances for treatment or were unable to drive felt that this would be much more beneficial than participants who had a short commute to receive treatment.

“*I had brain metastases from the minute I was diagnosed, I’m not allowed to drive. So getting to the hospital for the blood tests and the treatment etcetera becomes sort of a fairly major undertaking… So it means that my partner’s disrupted from work. I’m disrupted from work and then, you know, it adds to the time frame because I can’t just jump in the car and drive there*.” (M4)

##### Views on the balance of risk versus benefit changed over time

Most participants felt that their perception of personal risk and benefit changed over time. Many reported feeling fearful after their diagnosis, and as a result their primary focus was on controlling the cancer. Over time, participants whose cancer responded to treatment described a decrease in anxiety. Furthermore, most participants reported after initiating treatment they gained a better understanding about how treatment worked, and more insight into the implications of any side-effects that occurred. In these patients, their focus shifted from purely controlling the cancer to managing side effects and optimising HRQoL. This change in focus affected their personal assessment of risk and benefit and impacted their views about taking part in an ICI optimisation trial.

“*Yeah. It’s like priorities, isn’t it? The priority is to hit it hard. You’ll put up with the rest of it. And then there’s a point where actually the priority is now the side effects*.” (R14)

#### Theme 3: Pre-existing experience and beliefs about how treatment and trials work

Many participants had pre-existing beliefs about how treatment and trials work. This included the belief that more treatment would result in better disease control or that treatment should be tailored to the individual. Some participants also had the pre-existing belief that trials were a last resort when all other treatments had failed. These beliefs affected the acceptability of ICI optimisation trials.

##### Subtheme 1: Belief that more treatment is better

Most participants reported a pre-existing belief that the doses of treatment directly correlate with effect. Many participants translated their existing knowledge of other anticancer therapies, including chemotherapy and targeted therapies, into their understanding of ICI. Despite a presentation at the start of the FGD which outlined the mechanism of action of ICI and the evidence of overtreatment, most participants said they instinctively felt that higher doses of ICI would result in better disease control. This pre-existing belief made them less likely to agree to participate in an ICI optimisation trial, as they felt less treatment would be less effective at controlling the cancer.

“*Logically there’s no reason why more grunt [treatment] should work, but instinctively, that’s what you think*.” (R15)

“*Look, I can remember looking up at the bag and thinking, oh, there’s a little bit left in there and telling the nurse there’s a bit in there. Can you just wiggle it in? I want as much in me as possible*.” (R16)

Many participants reported that they found it difficult to understand how ICI worked, particularly when they were first diagnosed, which made it challenging to understand the logic for receiving less treatment.

“*Immunotherapy is still a really weird thing for people, I think to get to grips with and understand how it works… I really struggled with the concept of it at the beginning*.” (M4)

Most participants felt that patients would be more likely to consider participating in an ICI optimisation trial if they received clear communication from the oncology team explaining the scientific rationale. Importantly, this would need to include an explanation of the mechanism of action to emphasise that ICI do not result in the same dose-effect relationship we see with other treatments. A clear and appropriately pitched summary of the evidence supporting giving less ICI would be crucial.

“*But isn’t some of this kind of about education to people, you know, before they started immunotherapy, if it was properly explained how it worked, and the fact that the current dosing is random. I mean, that’s the thing they just threw everything at it as often as possible because that was, you know, it’s what you did with chemo. And so, if that was what was translated across before you started this, someone sort of explained, then people would start from a different place with it or would understand we’re all being completely over medicated*.” (M18 – previous clinical trial experience)

In contrast, some participants felt that they would not be able to process information about ICI and the logic of receiving optimised treatment at the time of diagnosis or during the early stages of their treatment as they were in a state of shock. Their decision making would be driven by emotions, and they may be too anxious to participate in an ICI optimisation trial.

##### Subtheme 2: Treatment should be personalised to the individual

Many participants had the belief that treatment dosing schedules should be personalised to the individual, for example doses based according to weight or other factors. Hypothetical trial designs that employed a personalised approached to optimise the dose and schedule of ICI were discussed. For example, trial designs which used therapeutic drug monitoring to measure ICI drug concentrations and formulate personalised treatment plans. Most participants felt this made intuitive sense and were very positive about taking part in a trial with a ‘personalised’ design.

“*Everybody is different, and everybody reacts differently to treatment. Everybody’s cancer is different. And so why should everyone be getting exactly the same standard treatment? And it makes sense to personalise it. And the less treatment you have to have is a benefit. So if you’re saying, the drugs are still in your system, still working, why give more when you don’t need them*?” (M19)

##### Subtheme 3: Trials are a last resort

Many participants who had not previously taken part in a clinical trial had the preconceived idea that trials were a last resort when all other treatments had failed. Consequently, some participants felt approaching patients about clinical trials early on in their diagnosis had the potential to be perceived negatively, the implication being that no other treatment options were available. Clearly communicating the nature of ICI optimisation trials and ensuring patients are aware of subsequent available treatments and trials should mitigate this concern. Providing general information on the role and nature of clinical trials may also help address the misconception of trials as a last resort.

“*Like the whole concept of a trial has something of a ring of last chance saloon about it doesn’t it?*” (M11)

“*I’m getting the impression that literally the first time that people talk about trials is when everything has failed, and you are out of options and you’re suddenly scarpering for what do I do next? And then what I find is in those groups when I’m talking to patients is they start asking strangers. Does anybody know about any trials?*” (C38)

## Discussion

This study demonstrates that although all participants found the concept of reducing treatment with ICI appealing, this was on the proviso that there was compelling data that this would be as efficacious as SOC treatment. Participants differed in their views of whether they would participate in an ICI optimisation trial.

One of the main aims of ICI optimisation trials is to improve patients’ HRQoL, assuming that a reduction in treatment visits would be desirable. However, this study showed that many patients find attending for treatment and clinic visits reassuring. The gap between patients’ needs and preferences, and physicians’ beliefs about them could be addressed through more involvement of patients and the public in the design and conduct of clinical trials. Designing trials that better meet patients’ needs and preferences may help improve recruitment to trials. In the case of ICI optimisation, potential benefits from reducing treatment and visits must be balanced against potential increased anxiety due to a perceived lack of clinical input and monitoring. To reduce this potentially negative psychological impact, trials with an early cessation or extended interval design could incorporate additional monitoring visits (which could be conducted virtually). Furthermore, additional psychological support may be beneficial for participants who are stopping treatment. Finally, trials should allow crossover from reduced intensity to SOC if the patient’s cancer progresses, and clearly communicating this at the outset of the trial to potential participants may aid recruitment.

Participants in this study evaluated the personal risks and benefits of participating in an ICI optimisation trial to inform whether they would take part. A primary concern was potential reduction in efficacy; therefore, views were influenced by prior response to treatment, with those who experienced the most benefit from ICI reporting more interest in these trials. The primary reason for enrolling in an ICI optimisation trial was a potential reduction in toxicity; participants with prior toxicities were more motivated to participate. A secondary factor was logistical, including reduced travel time, less impact on work, and the opportunity to spend more time with family and friends. Therefore, views were also influenced by the distance they had to travel for their treatment, whether they were able to drive, and employment status. Participants’ age, gender, whether they had dependents, and prior enrolment in a clinical trial, did not appear to influence their willingness to participate.

Many patients had the pre-existing belief that more treatment results in better disease control. Clearly communicating the scientific evidence supporting the hypothesis behind reducing ICI is essential to reassure patients that it is safe to participate. Many participants favoured clinical trials that offered personalised ICI treatment schedules. In designing ICI optimisation trials, teams should consider whether a personalised approach could be implemented. Finally, more general information about the role of trials may challenge the preconceived idea that clinical trials are a last resort.

Based on our findings, we propose several recommendations for ICI Optimisation Trials (developed with input from patient representatives). These are presented in Table 2. The first two recommendations centre around the importance of understanding and considering patient perspectives around ICI optimisation trials, through both patient and public involvement, and qualitative research. We also propose recommendations around the design of ICI optimisation trials. These include:

- exploring the potential for personalised approaches,
- allowing patients who have experienced toxicity to participate in trials,
- considering whether participants can revert to standard of care if their cancer progresses,
- providing support and monitoring to participants who are receiving less frequent or lengthy treatments,
- offering psychological support to those stopping treatment.

**Table 2:** Recommendations for ICI optimisation trials.

	Recommendation
All	Patient perspectives should be sought to help plan trial design/communication to improve recruitment strategies.
	Consider implementation of a qualitative sub study to investigate reasons why participants accepted or declined trial participation to refine trial recruitment strategies in a timely manner.
Trial design (all)	During trial development consider whether a personalised approach could be implemented (e.g. through therapeutic drug monitoring).
	Eligibility criteria should allow for inclusion of participants who have experienced toxicity, as these participants are often more motivated to participate.
	Consider whether it is possible for participants to revert to SOC if their cancer progresses.
Trial design of extended interval & early cessation trials	Ensure non-treatment monitoring visits are built into the trial protocol (remote monitoring is acceptable).
Trial design of early cessation trials	Consider offering additional psychological support if the participant stops treatment.
Communication	Include an appropriately pitched summary of the scientific rationale for ICI optimisation (including an explanation that research has demonstrated that there is not the same dose-effect relationship we see with other treatments).
	Clearly communicate both verbally and in the patient information sheet that participants on the experimental ‘optimised’ treatment schedules can revert to SOC if their cancer progresses.
	Highlight the trial visit schedule is not reduced despite fewer treatment visits and ensure patients have adequate opportunities to discuss any concerns with their clinical team.
	Provide general information on the role and nature of clinical trials to address the misconception of trials as a last resort.

The final set of recommendations relate to how we communicate with patients about ICI optimisations trials, including the scientific rationale (tackling the pre-existing beliefs around more treatment being better), and that trials are not always a last resort. Better communication on the way ICIs work, and the role of trials, would be beneficial for all patients for whom ICI is being considered, not just potential trial participants.

These study findings align with other research evaluating patients’ perspectives of optimising cancer treatment. Several studies have demonstrated that patients’ primary concern about participating in trials evaluating de-escalation of anti-cancer treatment is a potential reduction in efficacy [40, 41]. Whilst our findings were similar; understanding the scientific rationale for less treatment, consistent monitoring, and the ability to escalate treatment were factors that encouraged participation, a study conducted in the USA evaluating patient perspectives of optimisation of chemotherapy found that the lower financial burden associated with less treatment was also a key factor [41]. This was not reported as a factor for participants in this study, presumably as the UK’s healthcare service is publicly funded.

The participants in this study were all residing in the UK and therefore the results may not be transferable to a global population. Within the UK patients do not incur out of pocket expenses for ICI treatment, apart from travel costs and loss of earnings due to reduced ability to work. Patients living in countries with insurance-based healthcare systems or where they incur out-of-pocket expenses may place greater value on the lower financial toxicity that comes with less treatment. ICI optimisation trials in some countries may enable patients to access ICI treatment that would otherwise be inaccessible due to cost. This might make it easier to recruit participants to ICI optimisation trials in these countries.

This study aimed to be inclusive and accessible to a wide range of people, to ensure a range of perspectives were represented. Conducting FGDs online and in person allowed for the inclusion of a geographically diverse population, those who were too unwell to travel and people who were unfamiliar with using the online video conferencing platform. The data quality was similar in both the online and in person FGDs which has been demonstrated in previous studies [42, 43]. However, one limitation of this study was that there was a lack of ethnic diversity and there were no participants <35 or >75 years old. Therefore, this study may not have captured the views of these demographic groups.

As the FGD facilitators in this study (SM & HR) were both oncology clinicians, a potential power imbalance may have resulted in participants responding in more socially desirable ways and feeling unable to express certain views. To reduce this risk, patients who were known to either facilitator or treated at the hospital where they worked were not included in this study. The potential negative impact of having oncology clinicians as facilitators was felt to be offset by the fact that they were able to explore some topics in greater depth than would have been possible without the relevant clinical knowledge.

This qualitative study revealed that many patients found the concept of ICI optimisation appealing for the potential reduction in toxicity and convenience. Conversely some patients were concerned that receiving less treatment may result in a potential reduction in efficacy. Although conducting ICI optimisation trials will be challenging, there are potentially significant benefits for both patients and healthcare systems. Further research is needed to evaluate patient perspectives of ICI optimisation in early-stage cancer as treatment with ICI moves into the (neo)adjuvant setting for many cancers.

### Supplementary information

Supplementary materials

## Data Availability

The anonymised transcripts that support the findings of this study are available from the corresponding author, AS, upon reasonable request.
