# Supplementary material for: Hit it hard: qualitative patient perspectives on the optimisation of immune checkpoint inhibition
Source: Br J Cancer. 2024 Jun 17;131(3):515–23. doi: 10.1038/s41416-024-02756-x (PMC7616340; doi:10.1038/s41416-024-02756-x)
Supplement: Supplementary file 1 — Supplementary materials [file 41416_2024_2756_MOESM1_ESM.docx]

# Supplementary materials

## Index

1. Standards for Reporting Qualitative Research (SRQR) 2
2. Focus Group Topic Guide 5
3. Focus Group Presentation 9
4. Example of clustered codes 16
5. Optimal Cases 17

## Standards for Reporting Qualitative Research (SRQR)*

<http://www.equator-network.org/reporting-guidelines/srqr/>

**Title and abstract Page/line no(s).**

| **Title** - Concise description of the nature and topic of the study identifying the study as qualitative or indicating the approach (e.g., ethnography, grounded theory) or data collection methods (e.g., interview, focus group) is recommended | 1 |
| --- | --- |
| **Abstract** - Summary of key elements of the study using the abstract format of the intended publication; typically includes background, purpose, methods, results, and conclusions | 2 |

**Introduction**

| **Problem formulation** - Description and significance of the problem/phenomenon studied; review of relevant theory and empirical work; problem statement | 3 |
| --- | --- |
| **Purpose or research question** - Purpose of the study and specific objectives or questions | 3 |

**Methods**

| **Qualitative approach and research paradigm** - Qualitative approach (e.g., ethnography, grounded theory, case study, phenomenology, narrative research) and guiding theory if appropriate; identifying the research paradigm (e.g., postpositivist, constructivist/ interpretivist) is also recommended; rationale** | 4 |
| --- | --- |
| **Researcher characteristics and reflexivity** - Researchers’ characteristics that may influence the research, including personal attributes, qualifications/experience, relationship with participants, assumptions, and/or presuppositions; potential or actual interaction between researchers’ characteristics and the research questions, approach, methods, results, and/or transferability | 5, 14 |
| **Context** - Setting/site and salient contextual factors; rationale** | 4 |
| **Sampling strategy** - How and why research participants, documents, or events were selected; criteria for deciding when no further sampling was necessary (e.g., sampling saturation); rationale** | 4, 5 |
| **Ethical issues pertaining to human subjects** - Documentation of approval by an appropriate ethics review board and participant consent, or explanation for lack thereof; other confidentiality and data security issues | 5, 16 |
| **Data collection methods** - Types of data collected; details of data collection procedures including (as appropriate) start and stop dates of data collection and analysis, iterative process, triangulation of sources/methods, and modification of procedures in response to evolving study findings; rationale** | 4, 5 |
| **Data collection instruments and technologies** - Description of instruments (e.g., interview guides, questionnaires) and devices (e.g., audio recorders) used for data collection; if/how the instrument(s) changed over the course of the study | 5, Supplementary Materials |
| **Units of study** - Number and relevant characteristics of participants, documents, or events included in the study; level of participation (could be reported in results) | 5, 6 |
| **Data processing** - Methods for processing data prior to and during analysis, including transcription, data entry, data management and security, verification of data integrity, data coding, and anonymization/de-identification of excerpts | 5 |
| **Data analysis** - Process by which inferences, themes, etc., were identified and developed, including the researchers involved in data analysis; usually references a specific paradigm or approach; rationale** | 5, 6 |
| **Techniques to enhance trustworthiness** - Techniques to enhance trustworthiness and credibility of data analysis (e.g., member checking, audit trail, triangulation); rationale** | 5 |

**Results/findings**

| **Synthesis and interpretation** - Main findings (e.g., interpretations, inferences, and themes); might include development of a theory or model, or integration with prior research or theory | 6-12 |
| --- | --- |
| **Links to empirical data** - Evidence (e.g., quotes, field notes, text excerpts, photographs) to substantiate analytic findings | 6-12 |

**Discussion**

| **Integration with prior work, implications, transferability, and contribution(s) to the field** - Short summary of main findings; explanation of how findings and conclusions connect to, support, elaborate on, or challenge conclusions of earlier scholarship; discussion of scope of application/generalizability; identification of unique contribution(s) to scholarship in a discipline or field | 12-15 |
| --- | --- |
| **Limitations** - Trustworthiness and limitations of findings | 14 |

**Other**

| **Conflicts of interest** - Potential sources of influence or perceived influence on study conduct and conclusions; how these were managed | 16 |
| --- | --- |
| **Funding** - Sources of funding and other support; role of funders in data collection, interpretation, and reporting | 16 |

*The authors created the SRQR by searching the literature to identify guidelines, reporting standards, and critical appraisal criteria for qualitative research; reviewing the reference lists of retrieved sources; and contacting experts to gain feedback. The SRQR aims to improve the transparency of all aspects of qualitative research by providing clear standards for reporting qualitative research.

**The rationale should briefly discuss the justification for choosing that theory, approach, method, or technique rather than other options available, the assumptions and limitations implicit in those choices, and how those choices influence study conclusions and transferability. As appropriate, the rationale for several items might be discussed together.

**Reference:**

O'Brien BC, Harris IB, Beckman TJ, Reed DA, Cook DA. **Standards for reporting qualitative research: a synthesis of recommendations**. Academic Medicine, Vol. 89, No. 9 / Sept 2014 DOI: 10.1097/ACM.0000000000000388

## Focus Group Topic Guide

**Welcome and Introduction**

Good afternoon. My name is ……….. and I will be carrying out today’s focus group and I am the lead researcher for this study. I wanted to start by thanking you all for your time in taking part today.

I am a cancer doctor and I am currently taking time out of clinical practice to work in cancer research at a large clinical trials unit and I am completing a PhD which is looking at how we give immunotherapy treatment for cancer and some of the work I have been doing as part of this study will contribute to my thesis.

I would also like to introduce ………… who works with me at the research unit and who will be present for today’s session. Are you able to introduce yourself? (Co-facilitator introduction)

You have been invited to take part in this focus group because you have a diagnosis of melanoma/kidney cancer, and that type of cancer is sometimes treated with immunotherapy. Researchers would like to find out if we can find a way to give immunotherapy treatments differently so that we can reduce side effects and improve quality of life for patients. But to run these we really want to understand patient views on how best to do this. We have been running a series of focus groups with patients with kidney cancer and melanoma.

For today’s discussion, we are interested in hearing from you about what your opinions are on reducing the amount of immunotherapy for patients with cancer and how to talk to patients about participating in a trial where less immunotherapy is given. There are no right or wrong answers here today. We want to understand different experiences and views of this approach and we don’t all need to reach a consensus. Please be open with us.

I will start with a few bits of housekeeping for the session today. The focus group will take approximately 90 minutes. We will start with a short presentation to set the scene. The rest of the focus group will be a discussion between the group. Please could you check your phones are on silent.

There will be a comfort break halfway through. If anyone needs to leave the focus group at any point, please do so.

Can I ask everyone to introduce themselves to the group? Please let us know your name and where you are from. If you feel comfortable to do so you can also tell us whether you have ever received immunotherapy or taken part in a clinical trial before. You do not have to do this. This part of the session is not being recorded.

*Introductions*

Thank you. Before we start, I want to ask you not to share what other people have said during the focus group today. We will not share your responses with your doctors or anyone outside the research team. We may use data, without your name or other information by could identify you, in quality improvement reports and publications. We would refer to you as ‘participant 1, participant 2 etc’. We will be recording on the laptop on Teams and on a dictaphone as a backup in case one recording doesn’t work. Can I check that everyone is happy for me to record our focus group so I can best capture our discussion?

Does anyone have any questions before we begin?

Answer questions.

**RECORDING STARTS**

**Rationale for reduced intensity ICI**

I will briefly talk to you about why we believe we would like to test reducing the amount of immunotherapy that we give to patients.

Presentation:

- Slides on rationale for reducing the intensity and why this is important
- Aims of the focus group today

Do you have any questions before we begin?

**Group Discussion ONE Views of reducing intensity of ICI and views on participating in a clinical trial investigating reducing intensity of ICI**

**Reduced Frequency**

- What would appeal to you about taking part in this kind of trial?
  - Prompts: Reducing side effects of treatment, logistical benefits (ability to work/care for children/travel), having time away from cancer centre
  - Does the recent covid pandemic affect decision?
- What would put you off taking part in this kind of trial?
  - Prompts: fear of cancer growing, worry about regret, lack of interest in not getting something ‘standard of care’, focus on doing less instead of more

**Reduced Duration**

1. What would appeal to you about taking part in this kind of trial?

Prompts: Reducing side effects of treatment, logistical benefits (ability to work/care for children/travel), having time away from cancer centre

- - Does the recent covid pandemic affect decision?

1. What would put you off taking part in this kind of trial?

Prompts: fear of cancer growing, worry about regret, lack of interest in not getting something ‘standard of care’, focus on doing less instead of more

**Reduced Dose**

- What would appeal to you about taking part in this kind of trial?

Prompts: Reducing side effects of treatment, logistical benefits (ability to work)

- What would put you off taking part in this kind of trial?
  - Prompts: fear of cancer growing, worry about regret, lack of interest in not getting something ‘standard of care’, focus on doing less instead of more

**BREAK**

**Show slide of all three trials.**

I would now like you to look at the following slide and consider if you were going to take part in one of these trials, which one would appeal the most to you? Please could you write down your answer on a piece of paper and when we come back from the break we will come around and discuss why you chose that option.

**Immunotherapy Drug A is normally given at a dose of 400mg every 4 weeks for 2 years.**

Trial A: Tests giving 2**00mg** (half the dose) every 4 weeks for 2 years

Trial B: Tests giving 400mg (the usual dose) **every 8 weeks** for 2 years

Trial C: Tests giving 400mg (the usual dose) every 4 weeks for **1 year**

**I would not want to take part in any of these trials**

**BREAK**

**Ask people why they chose particular trial.**

- If less immunotherapy was shown to be just as effective in a clinical trial, how would you feel about receiving less treatment (either lower dose, less often or shorter amount of time) as part of routine cancer treatment?
- Some research studies are trying to develop immunotherapy drugs which can be given as a simple injection under the skin by a trained nurse (a bit like an insulin injection for diabetes). This might mean that you could have your treatment outside of hospital e.g. in a local clinic. Would this change your views on what we have already discussed?
- How would you feel about having tests (such a blood tests or scans) which could be used to guide if and when you have your next immunotherapy drug (i.e. personalise your individual cancer treatment schedule)?

**Group Discussion THREE – Preferred Language and approach**

Go back to trial – randomising after 3 months.
When would you recommend discussing a trial like this with patients? When diagnosed, before starting immunotherapy, after a few weeks of starting immunotherapy?

How would you like to be communicated with about this trial?

- Discussion with medical team (doctor, research nurse/both)
- Written information
- Website
- Video
- Podcast
- Other

How would it be best to sequence this information?

We are interested in what words we can use to describe reducing the intensity. What do you think of the following words? (positive/negative feeling)

**Prompts:**

- 1. De-escalation
  2. De-intensification
  3. Reduced intensity
  4. Optimisation
  5. Personalisation
  6. Lowest effective dose

Have any other words sprung to mind that would be better?

(Any specific words about reduced dose, less often, shorter time?)

We are aware that some people may be nervous of taking part in a trials like these because they will be worried about having less treatment. Have you got any recommendations about the best way to discuss this with patients?

**Wrap up**

Thank you very much for taking the time to participate in the focus group today and thank you for contributing. We will be emailing you the vouchers for taking part over the next couple of days. We will be writing up a transcript and analysing what was said.

Before we finish, I would like to remind everyone not to share what other people have said during the focus group today.

Does anyone have anything they would like to add before we finish today?

Thank you. **Stop Recording**

## Focus Group Presentation


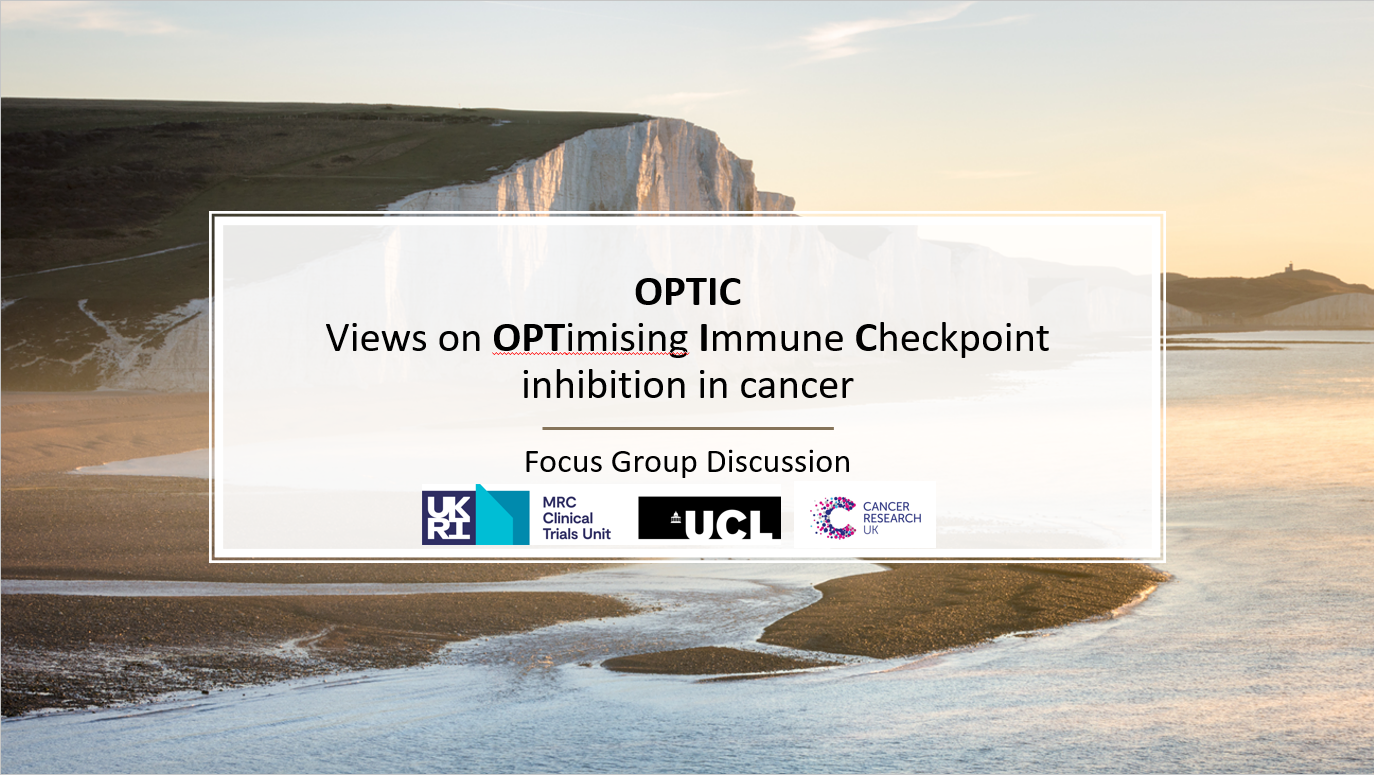


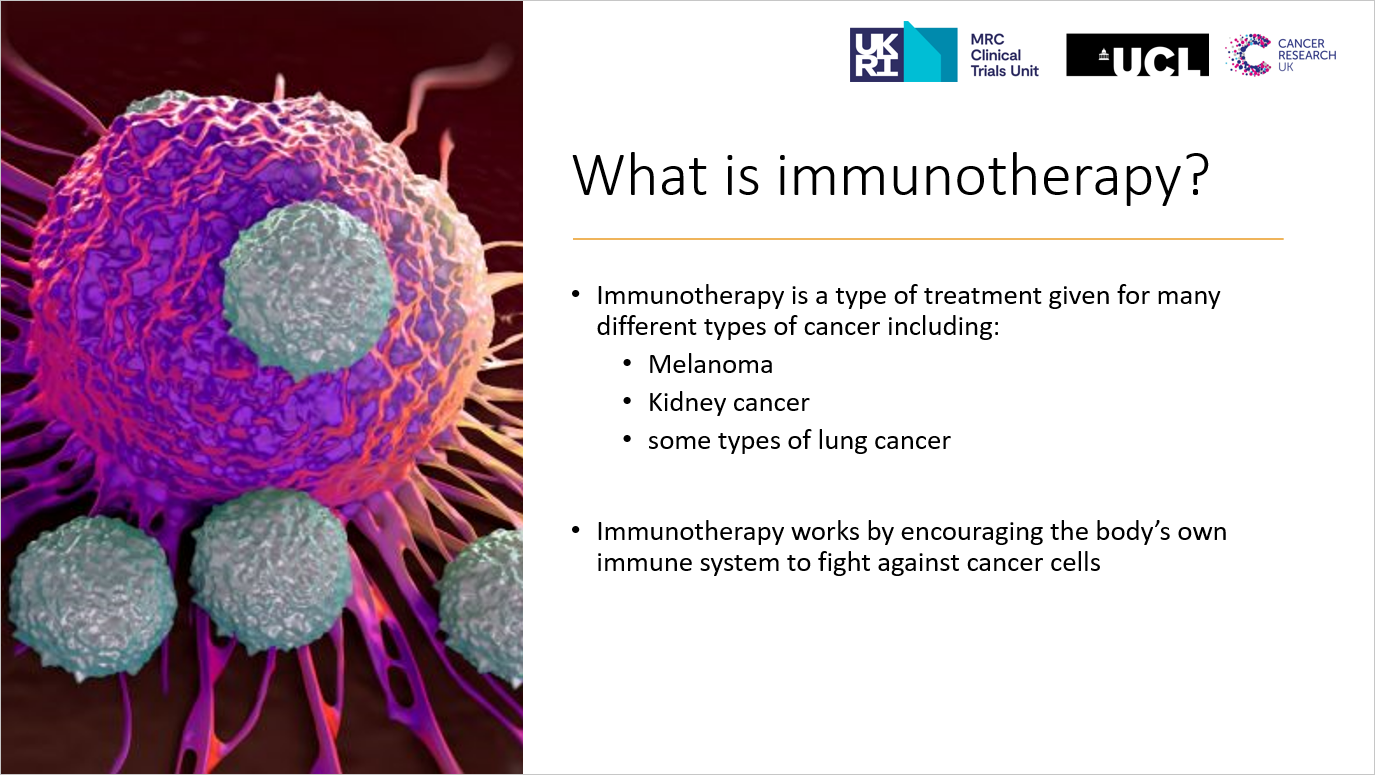


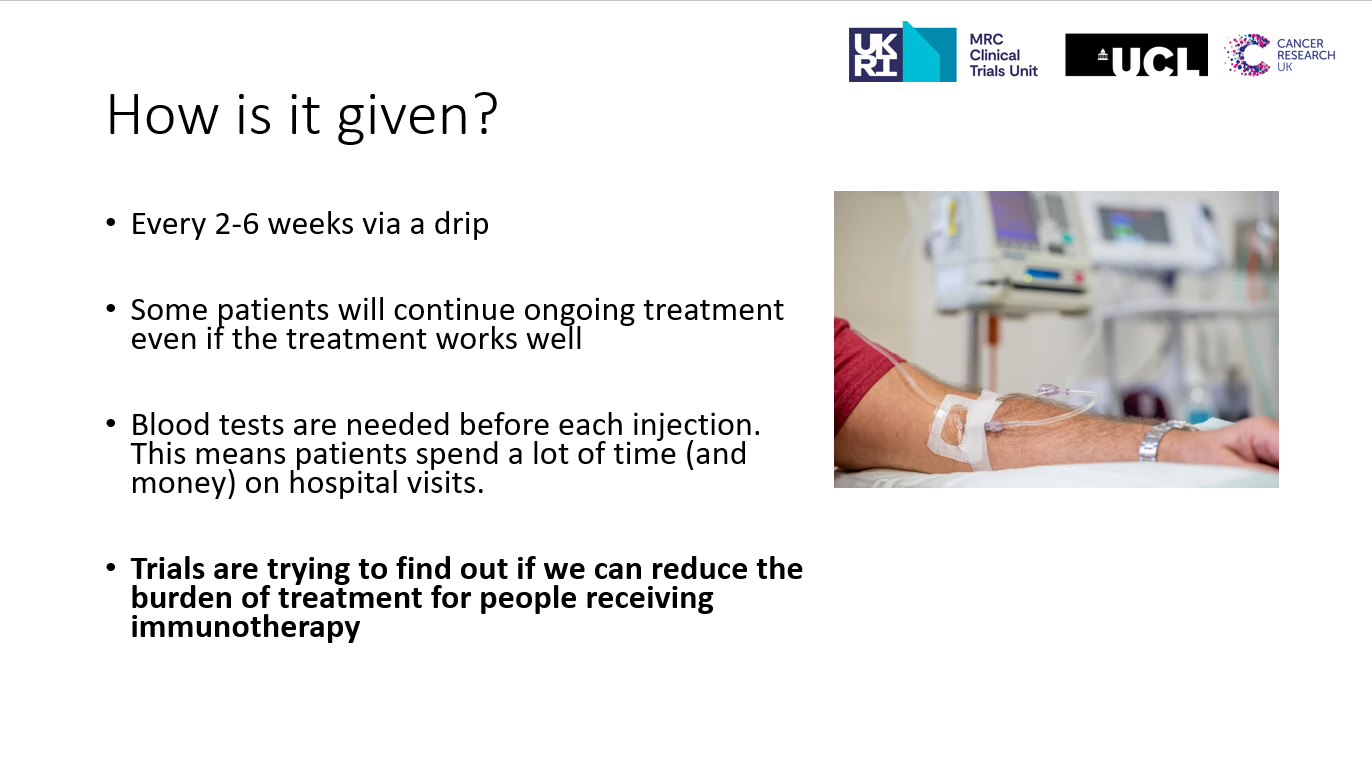


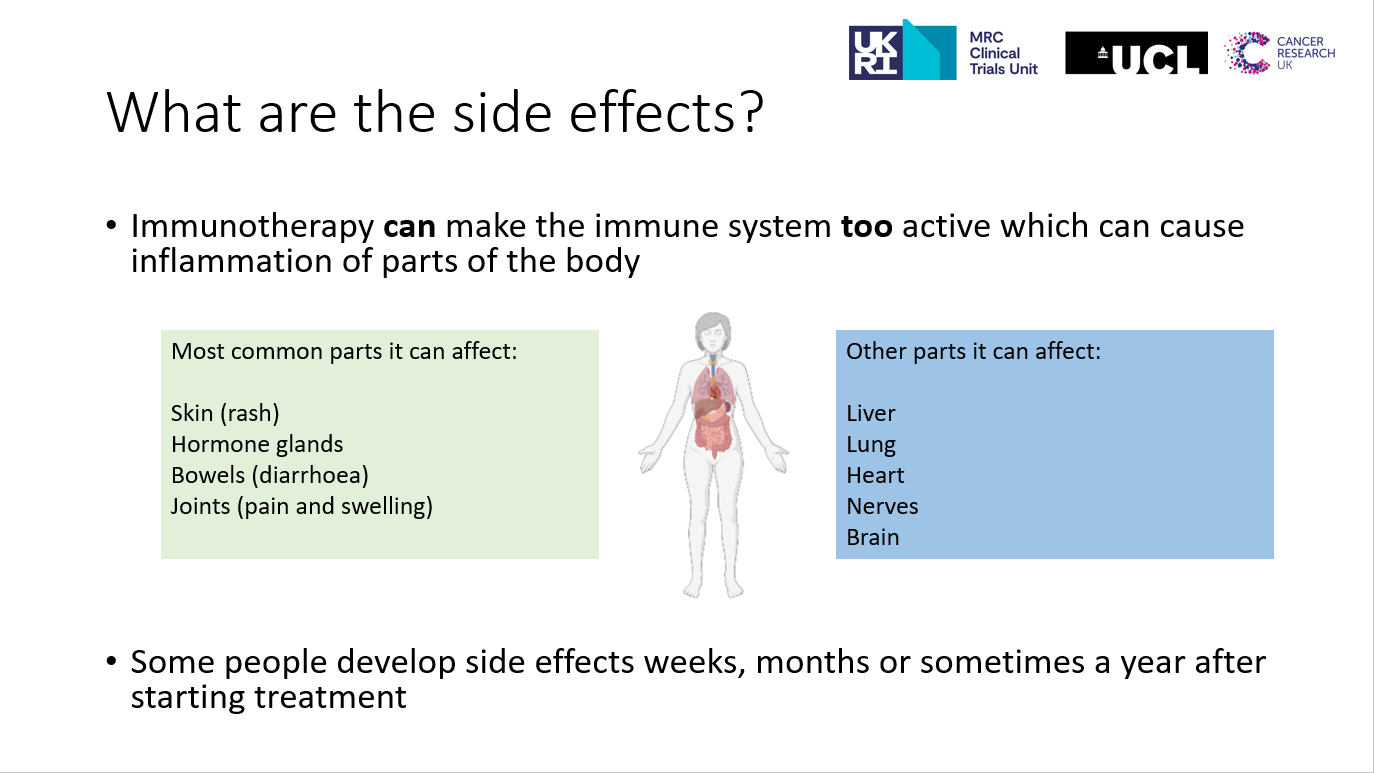


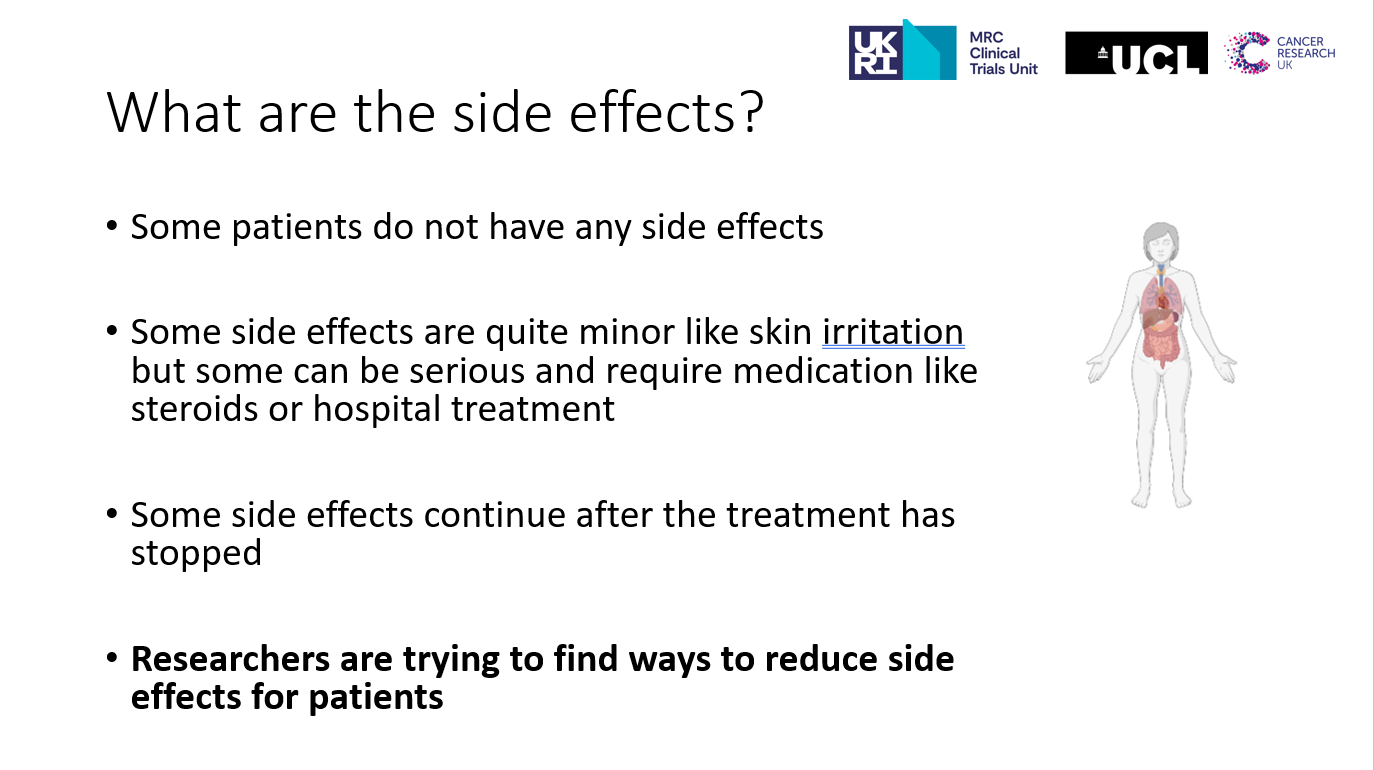


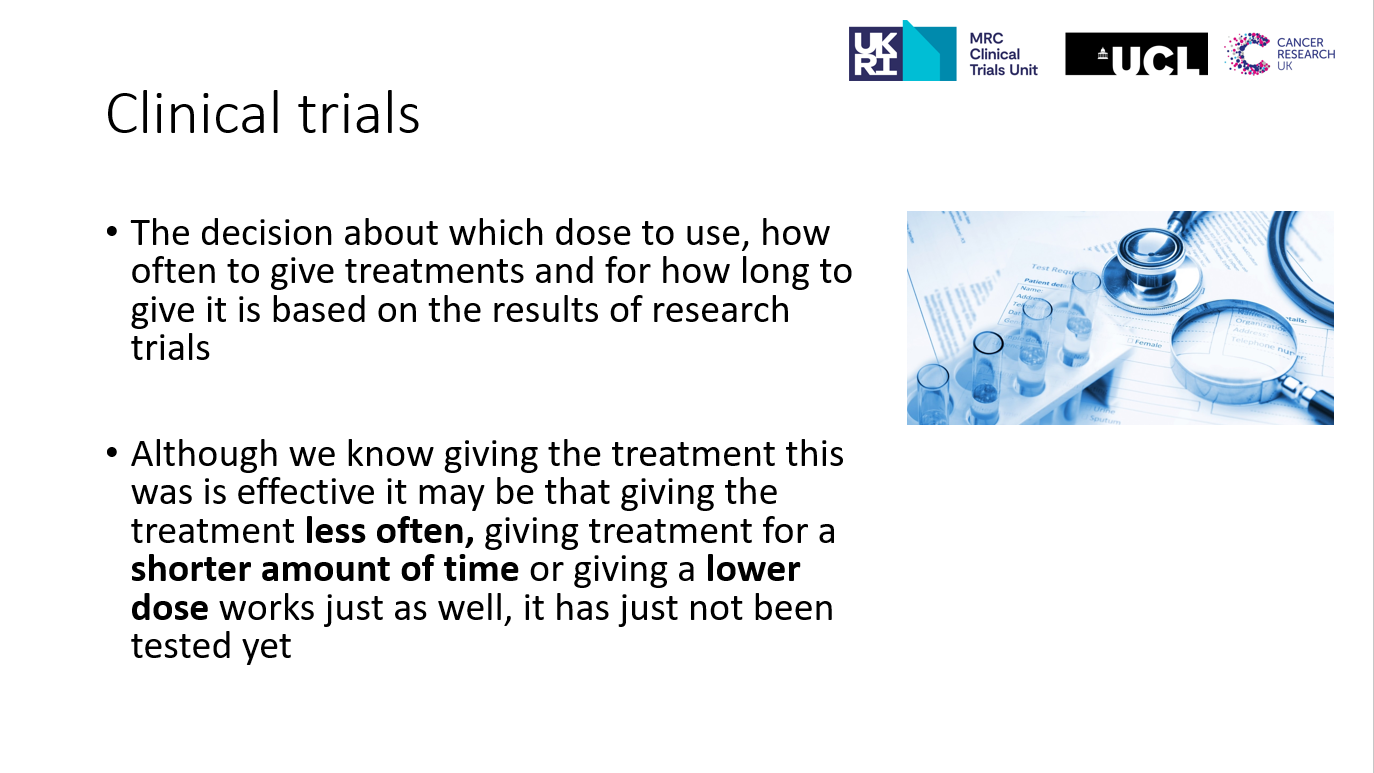


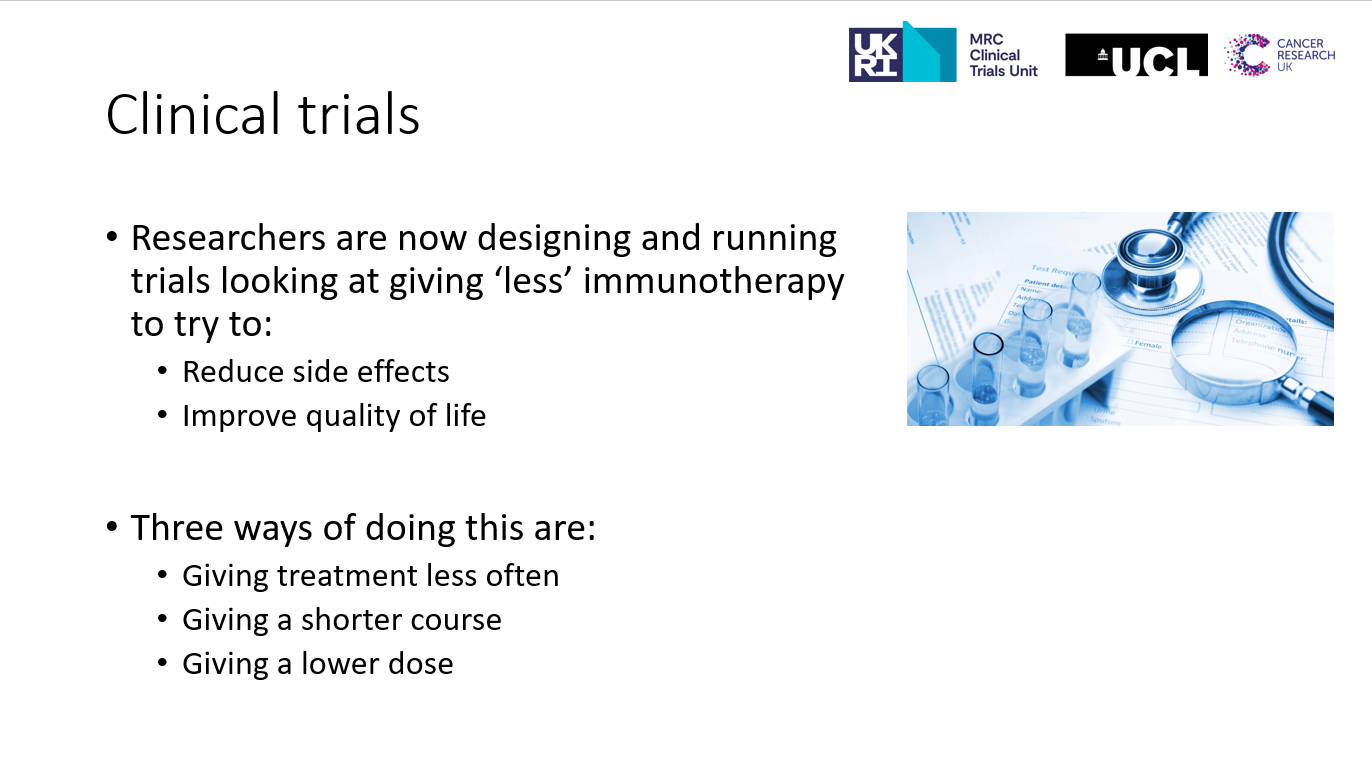


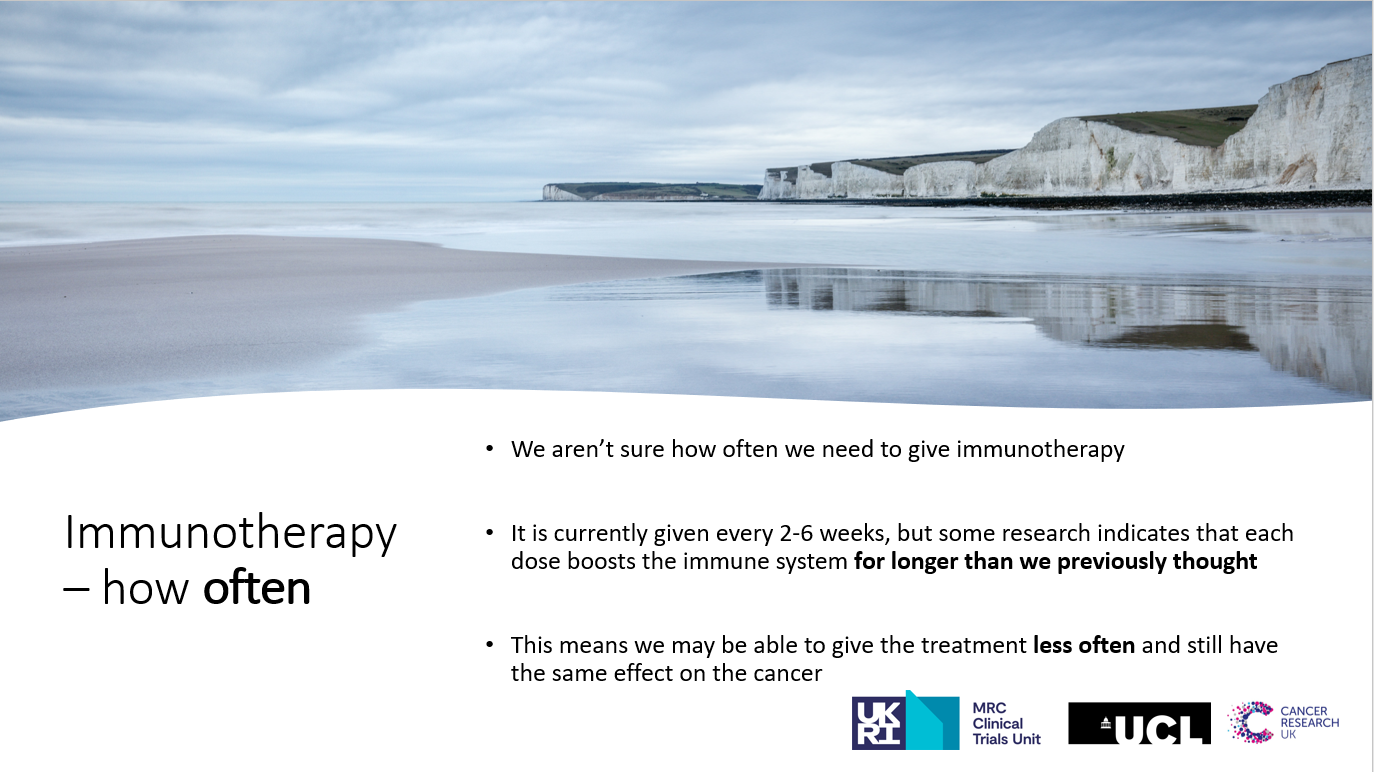


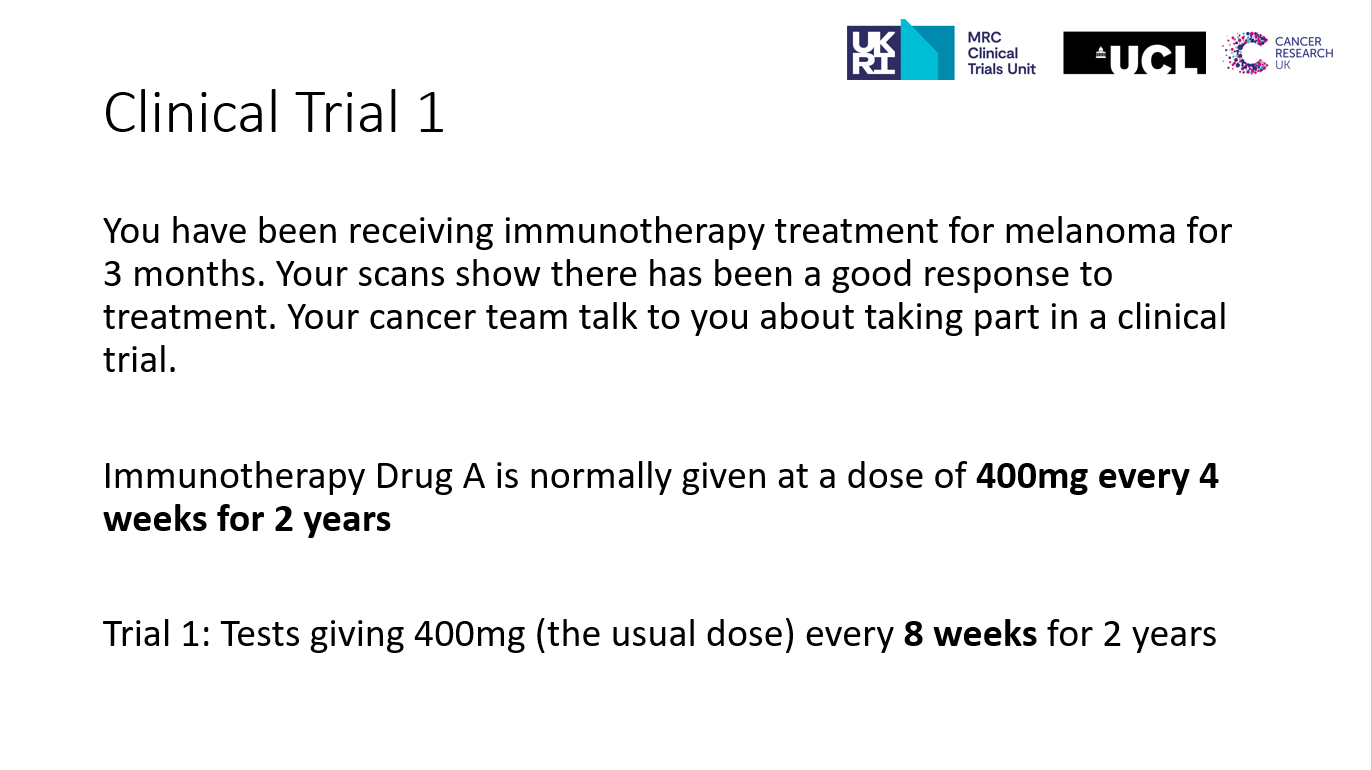


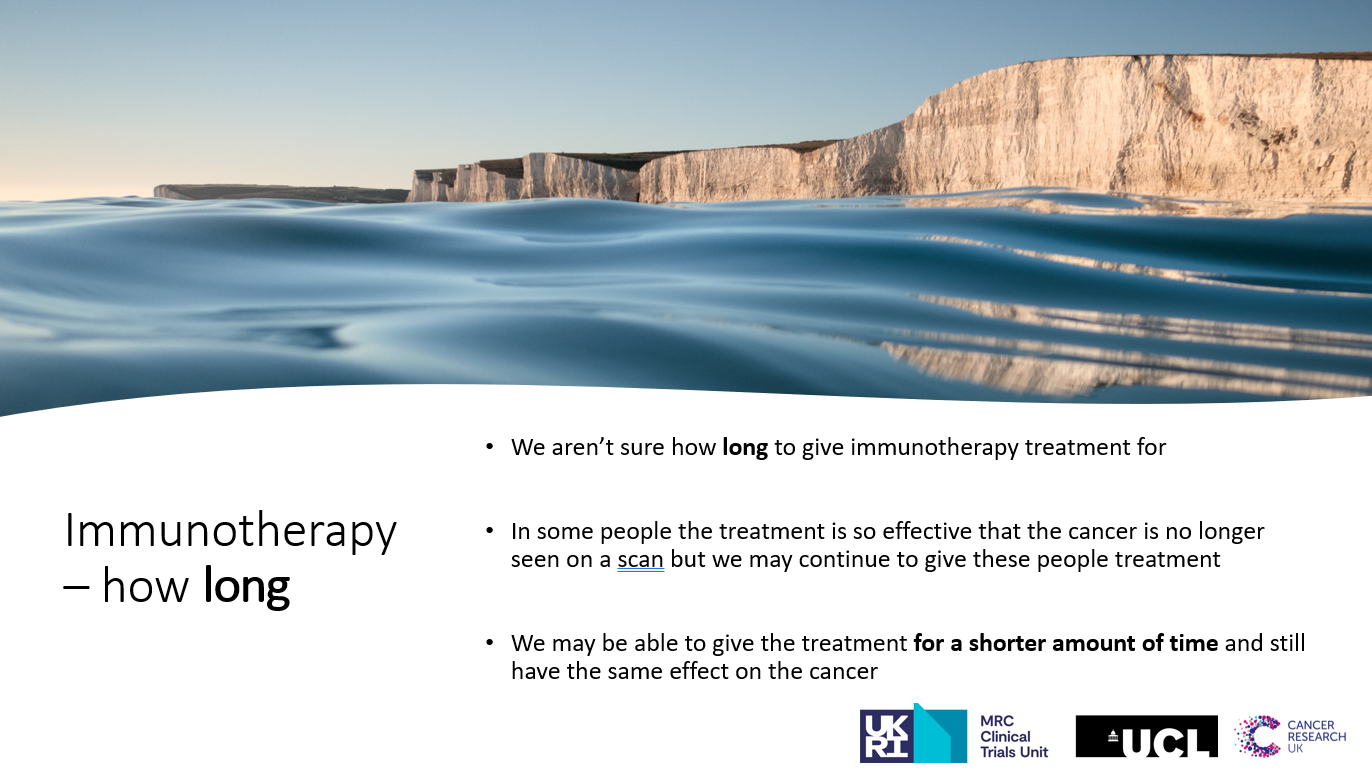


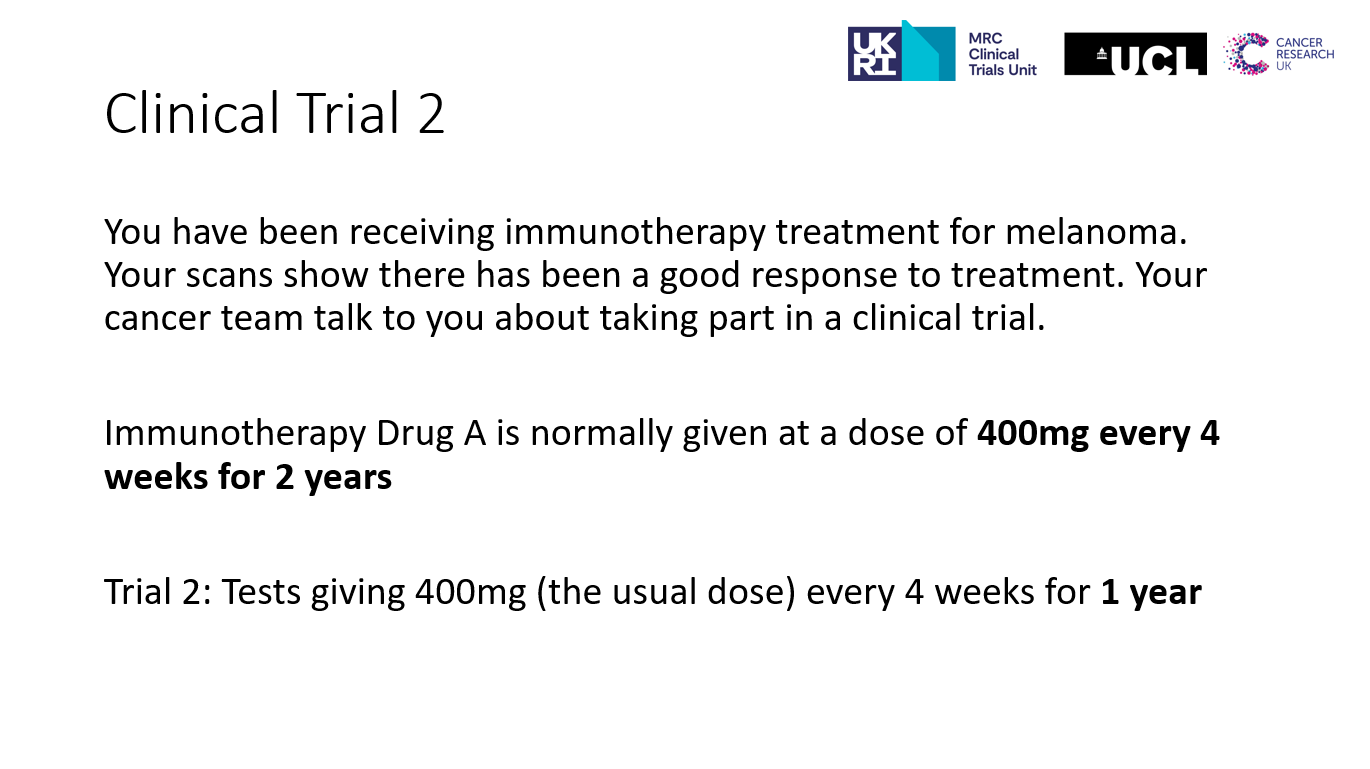


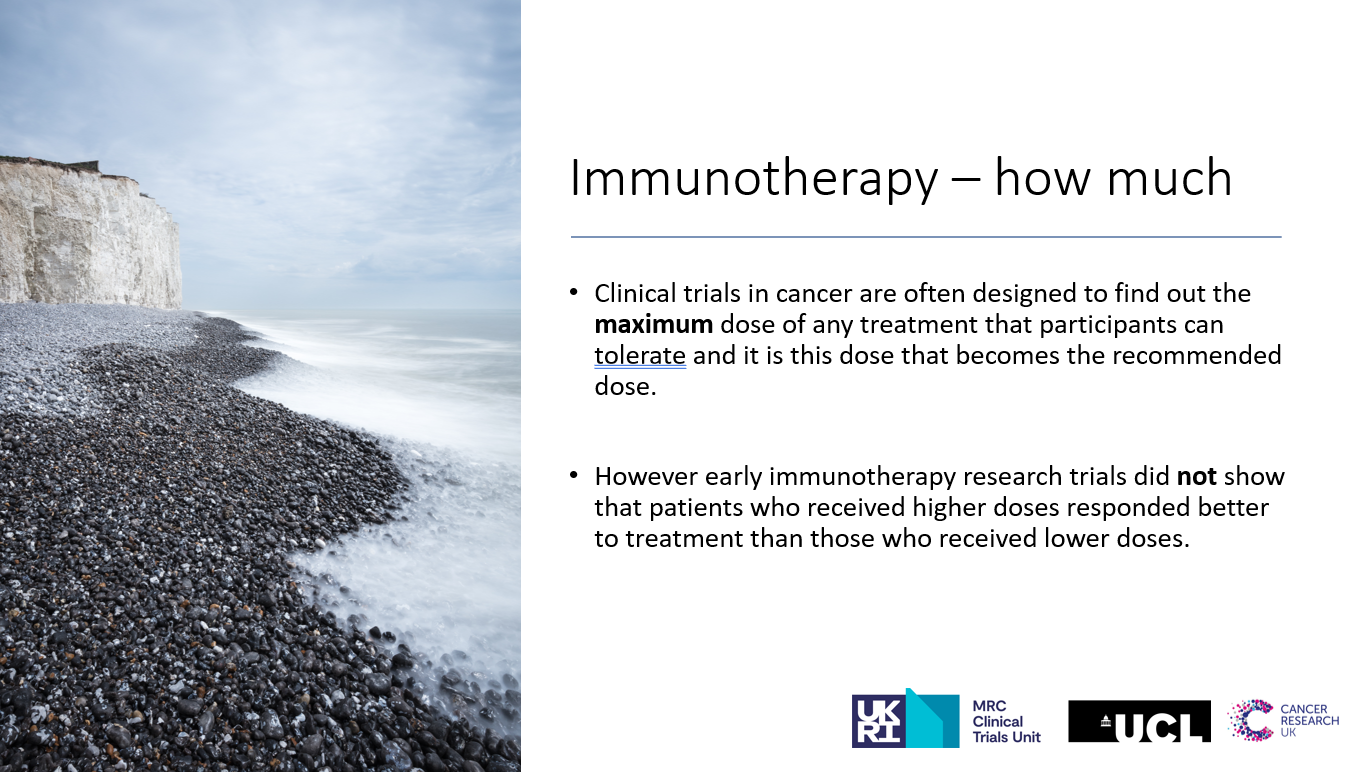


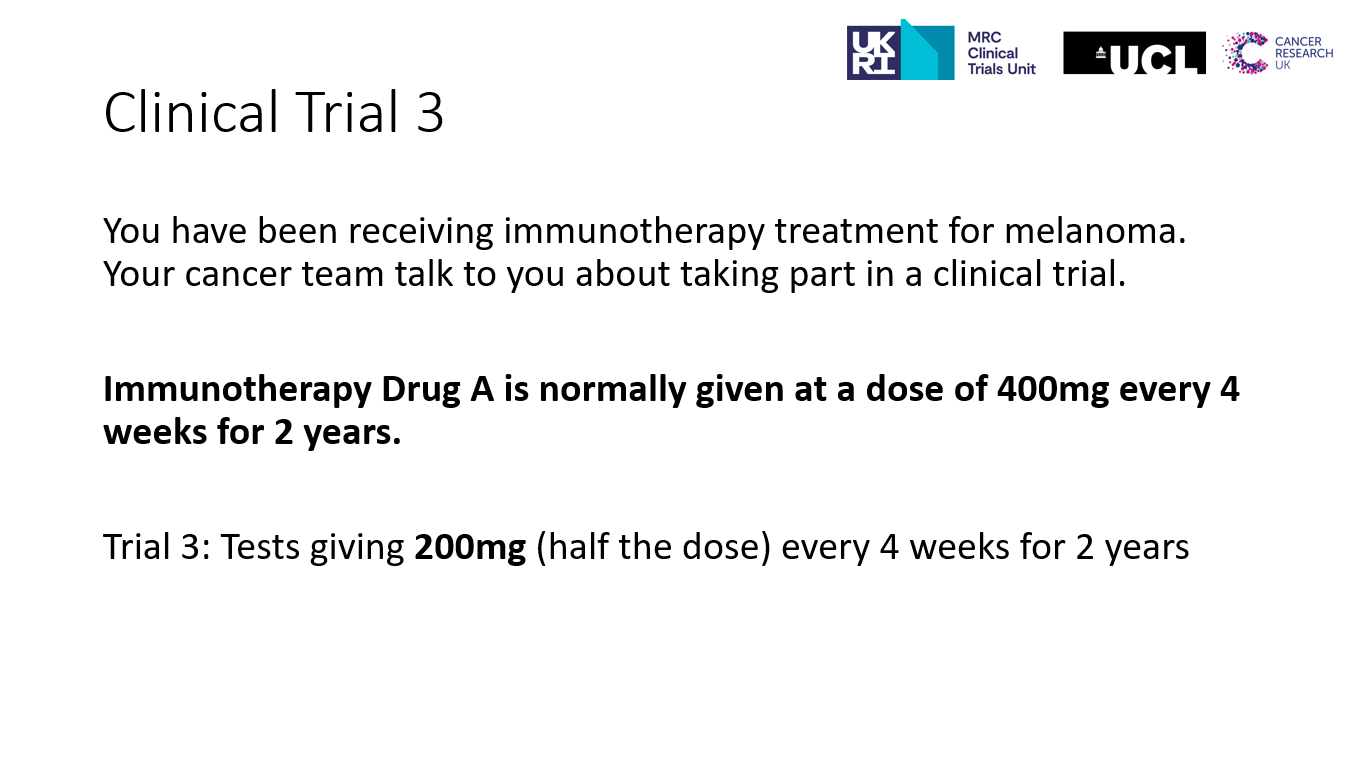

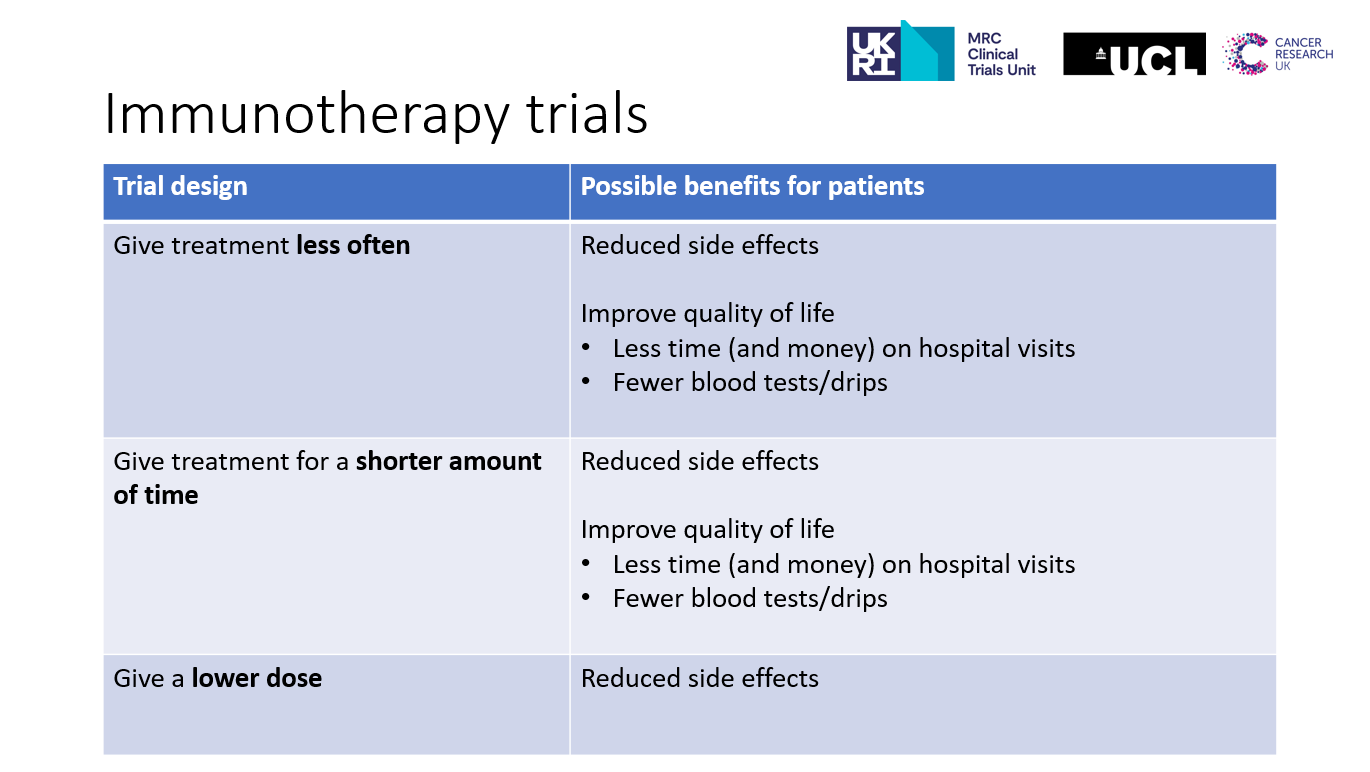


## Example of clustered codes

The diagram below shows an example of clustered codes in NVivo 12.


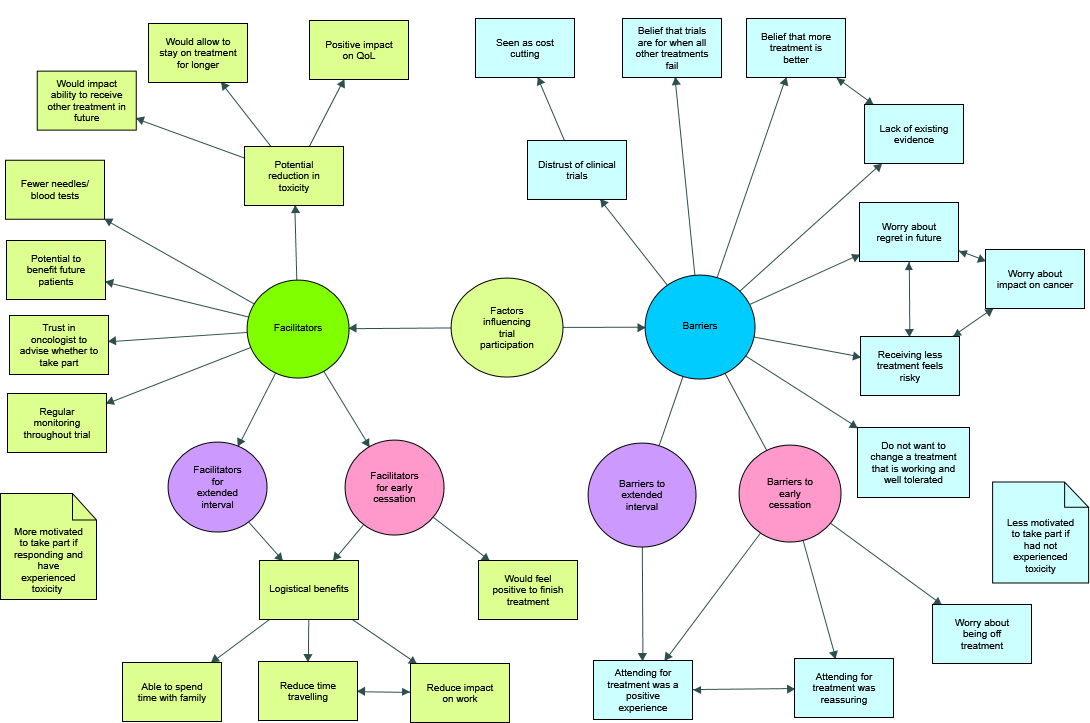


## Optimal Cases

An ideal type analysis was performed to explore whether certain participant characteristics influenced their views on ICI optimisation. Three ‘optimal cases’ were identified. For the group which felt negatively about ICI optimisation trials there was no clean example that typified all characteristics of the group.

### Case 1: Positive about participating in an ICI Optimisation Trial

M29 was a man aged 46-55 who was diagnosed with Stage IV melanoma. He was actively working throughout much of his treatment. He received combination ipilimumab/nivolumab followed by nivolumab maintenance with a total treatment duration of over 2 years. He experienced high grade toxicity during his induction treatment and continued to experience low grade toxicity during the maintenance phase. He had a complete response early during his treatment and was keen to stop ICI but was unable to as NHS guidelines stipulated that he would need to receive the full course of ICI (2 years) so that he could be retreated in the event of progression in future.

*“It really felt like I was just treading water. I don't really feel like there was progress made when I was doing the single treatments for so long. Nothing seemed to happen. It just seemed to sort of give me side effects for no real gain for such a long period after.”*

*“I honestly felt that two years was arbitrary, and you know, like the only reason I was doing it was to fulfil it on track so that they would treat me again if I got sick again.”*

In addition to finding the potential reduction in toxicity with receiving less treatment appealing he also felt an ICI optimisation trial would provide the opportunity to spend time away from the hospital to be with his family.

*“You are continually at a hospital somewhere, you know. Yeah. And for so long, it’s a real undertaking.”*

He had a long travel time to receive treatment (1-2 hours) and was working during most of his maintenance treatment. He felt there would be logistical benefits of taking part in an ICI optimisation trial, including reducing travel time and the impact on work.

He felt taking part in an ICI optimisation trial would improve his quality of life, particularly extending the interval between treatments, or stopping treatment.

*“That would have appealed - a shorter course. Once I’d had the positive result early on, I don't really feel like had to go on for two years. But for me, because of the delay in treatments and the rounds, it was about 27 months all in all. And the other thing is, you mentioned going from maybe four weeks cycles to you know more like 12 weeks and all the rest of it. That would obviously have improved my quality of life immensely at the time.”*

### Case 2: Unsure about participating in an ICI Optimisation Trial

R3 was a man aged 46-55 who had been diagnosed with localised RCC several years ago for which he underwent a nephrectomy. He recurred with metastatic disease a few years later and received combination treatment with nivolumab/ipilimumab followed by nivolumab maintenance therapy. His cancer progressed while on nivolumab maintenance and was subsequently treated with tyrosine kinase inhibitors. R3 had experienced toxicity because of his ICI treatment, including hypoadrenalism and transaminitis which required treatment with steroids. He reported that at the time he was diagnosed he would have wanted as much treatment as possible, but that his views changed over time. After developing toxicity and he would have considered ICI optimisation if this may potentially reduce toxicity.

*“At the time [of diagnosis] I would want to throw as much clout and firepower at it as I think will give it a chance. So you know without the benefit of hindsight and some of the problems I had, you know, I'd say throw everything at it. I don't mind the risk because the benefit, you know, massively outweighs you know what's happened. So you want that chance, don't you?”*

*“Yeah if there's less chance of this adrenal issue, which affects a lot of people, then that would be quite an important part of my decision, really. I think I, and a lot of people, would probably then opt for the less frequent option.”*

R3 was working during ICI treatment and lived a short distance from the hospital (<15 minutes). He did not find attending for treatment regularly had a negative impact on him. Therefore, the logistical benefits would not play a part in his decision making of whether to take part in an ICI optimisation trial.

*“I'm, I don't know, 10/15 minutes from [name of] Hospital. When I had immuno[therapy] especially, I found a chance to actually talk to other cancer patients. I never found it too dreadful.”*

### Case 3: Negative about participating in an ICI Optimisation Trial

M30 was a woman aged 36-45 who was diagnosed with Stage IV melanoma and had received combination ipilimumab/nivolumab. After three cycles of combination treatment she developed toxicity, which required treatment with IV steroids. This resulted in treatment delays prior to establishment on maintenance nivolumab. She tolerated maintenance treatment well with minimal side effects other than fatigue.

*“I managed 3 doses of the double [ipilimumab and nivolumab] and then had some very nasty like arthritic side effects and then anterior uveitis. But luckily I was able to restart and I'm seems to be tolerating the single really well, but I remember how frightened I was during that gap when they kept saying no, we're still not happy to give you treatment. I was on IV steroids. So until I could get that dose down, I knew I couldn't have my liquid gold.”*

At the time that M30 participated in this study, she had received a year of treatment. Although she had responded to ICI, she had not had a complete response, and this was her primary concern about receiving ‘less treatment’.

*“Maybe I'd feel very different if they said you're NED (no evidence of disease) right now. We feel confident that, OK, let's crack on, but not while the tumours are there, no, just get them out of my body.”*

She was fearful about reducing her treatment and the potential negative impact on the cancer. She felt this was much more important to her than the impact of potential lifelong side effects.

*“Yes, that would be my absolute fear. I'd rather deal with the lifelong side effects than you know, be 6 feet under.”*

M30 had a short travel time for treatment (<30 minutes) and had not been working throughout the course of treatment. Therefore, she felt the logistical benefits would not be relevant to her personal decision-making process about whether to take part.

*“I'm not working…….* *I guess I haven't had to do any juggling. I don't have children. I live about a half hour walk from my hospital, so I kind of take myself there. I don't have to deal with traffic or parking costs or the congestion charge in the city. You know, all of those things which I know can really add stress to other patients.”*
